# *In vivo *characterisation of the Golgi matrix protein giantin: linking extracellular matrix secretion and cilia function

**DOI:** 10.1186/2046-2530-4-S1-P38

**Published:** 2015-07-13

**Authors:** D Bergen, D Asante, N Stevenson, P Verkade, C Hammond, D Stephens

**Affiliations:** 1 School of Biochemistry, University of Bristol, Bristol, UK; 2 School of Physiology and Pharmacology, University of Bristol, Bristol, UK; 3 Wolfson Bioimaging Facility, University of Bristol, Bristol, UK

## 

The C-terminal anchored golgin giantin (*golgb1*) plays a crucial role in Golgi structure and acts as a tether for COPI vesicles at the *cis*-and-*medial *Golgi. Recently, our lab has shown that in addition to its known roles in membrane trafficking, giantin is required for ciliogenesis *in vitro*. A functional knockout of giantin exists. The osteochondrodysplasia rat, that arose from a spontaneous insertion in the *golgb1 *gene. Homozygous embryos depicted severe craniofacial defects and oedema, caused by defects in extracellular matrix composition. However, *ex vivo *cultured skin fibroblasts from these animals showed no major ciliogenesis defects. To further elucidate the role of giantin in extracellular matrix and cilia function we have used morpholino knockdown in the zebrafish. Knockdown resulted in a curly tail down and tail tip up phenotype with severe cardiac oedema, hydrocephalus, and craniofacial defects in the pharyngeal craniofacial structures. Immunohistochemistry revealed that collagen-2 expression was altered in an ectopic fashion in these structures. Furthermore, defects in cilia function were observed. In the neural tube cilia number was reduced and visible cilia were longer. Additionally, morphants showed randomisation of the heart position, suggesting left-right patterning is affected. This work is strongly implying a dual role for giantin in extracellular matrix deposition and cilia function.

